# Supplementary material for: Design and Simple Assembly of Gold Nanostar Bioconjugates for Surface-Enhanced Raman Spectroscopy Immunoassays
Source: Nanomaterials (Basel). 2019 Nov 4;9(11):1561. doi: 10.3390/nano9111561 (PMC6915668; doi:10.3390/nano9111561)
Supplement: Supplementary file 1 [file nanomaterials-09-01561-s001.pdf]

nanomaterials

MDPI

Supplementary Information

Article

Design and simple assembly of gold nanostar bioconjugates for Surface-Enhanced Raman Spectroscopy immunoassays

Maria João Oliveira [1,2], Miguel P. de Almeida [3], Daniela Gomes [2], Elvira Fortunato [2], Rodrigo Martins [2], Eulália Pereira [3], Hugh J. Byrne [4], Hugo Águas [2,*], and Ricardo Franco [1,*]

[1] UCIBIO, REQUIMTE, Departamento de Química, Faculdade de Ciências e Tecnologia, Universidade NOVA de Lisboa, 2829-516 Caparica, Portugal; mj.oliveira@campus.fct.unl.pt (M.J.O.)

[2] CENIMAT-I3N, Departamento de Ciência dos Materiais, Faculdade de Ciências e Tecnologia, FCT, Universidade Nova de Lisboa, 2829-516 Caparica, Portugal; Daniela.gomes@fct.unl.pt (D.G.); emf@fct.unl.pt (E.F.); rfpm@fct.unl.pt (R.M.)

[3] REQUIMTE/LAQV, Departamento de Química e Bioquímica, Faculdade de Ciências da Universidade do Porto, 4169-007 Porto, Portugal; mpda@fc.up.pt (M.P. de A.); eulalia.pereira@fc.up.pt (E.P.)

[4] FOCAS Research Institute, Technological University Dublin, Kevin Street, Dublin 8, Ireland. Hugh.Byrne@tudublin.ie (H.B.)

* Correspondence: ricardo.franco@fct.unl.pt (R.F.); hma@fct.unml.pt (H.A.)

1 Diameters and concentration for AuNPs and AuNSs synthesized

Gold nanoparticle samples, spheres (AuNPs) and stars (AuNSs), were characterised by UV-Visible spectroscopy, DLS and XRD. Table S 1 summarises the diameter obtained from the different techniques.

For AuNPs, the UV-Vis spectrum revealed a LSPR band used to determine the average size and molar concentration of spheres (12.0 ± 0.2 nm and ≈14 nM).[1] The mean size of AuNPs obtained by XRD and DLS measurements were 13 nm, which is in agreement with the obtained by UV-Vis spectrum. For AuNSs the same techniques were applied. The UV-vis spectrum was used to determine the concentration in nanomolar of AuNSs achieving a concentration of 0.6 nM.[2] The DLS showed a diameter of ≈70 nm which is in agreement with the literature with the same synthesis method.[3]

XRD analysis allowed to confirm the crystalline nature of AuNPs and AuNSs (Figure S 1). The diffraction pattern revealed peaks at 38.17º, 44.51º, 64.65º and 77.69º and finally, 81.87º. The first four correspond to (111), (200), (220) and (311) Bragg's reflection of crystalline metallic gold, respectively. This profile is in good agreement with reference to the unit cell of face centre cubic (fcc) lattice of metallic gold (JCPDS File No. 00-001-1174) with a lattice parameter of a = 4.0640 Ǻ and with a space group of Fm-3m. The broadening of Bragg's peaks indicates the formation of nanoparticles. The crystalline structure of both gold nanoparticles, spheres and stars, is cubic since the (111) is the dominant orientation as previously reported.[4], [5] The mean size of AuNPs was calculated using the Debye–Scherrer's equation achieving 13 nm (Table S 1). Nevertheless, to our knowledge, an appropriate modelling is still needed for anisotropic nanoparticles.

Table S 1 - Summary of the obtained diameters and concentration for AuNPs and AuNSs synthesized.

Nanoparticle type	λ_{LSPR} (nm)	Diameter (nm)			Concentration (nM) by UV-Vis spectroscopy
		UV-Vis spectroscopy	DLS	XRD	
**AuNP**	519	12.0 ± 0.2	13 ± 0.3	13	13.83 ± 0.22
**AuNS**	708	-	67.9 ± 0.4	-	0.60 ± 0.01

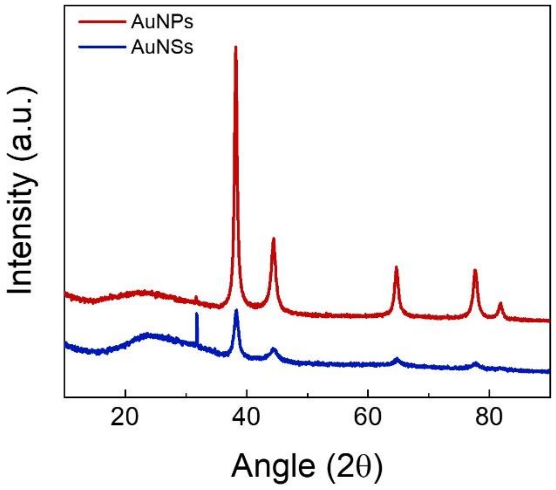

Figure S 1 – XRD pattern of as-prepared sphere and star gold nanoparticles.

2 Reproducibility between batches of newly synthesized AuNSs

The uniformity was studied using UV-Vis spectroscopy and Dynamic Light Scattering (DLS) (Figure S 2). The results provided by both techniques used to test the AuNSs solution homogeneity confirmed the reproducibility of the synthesis.

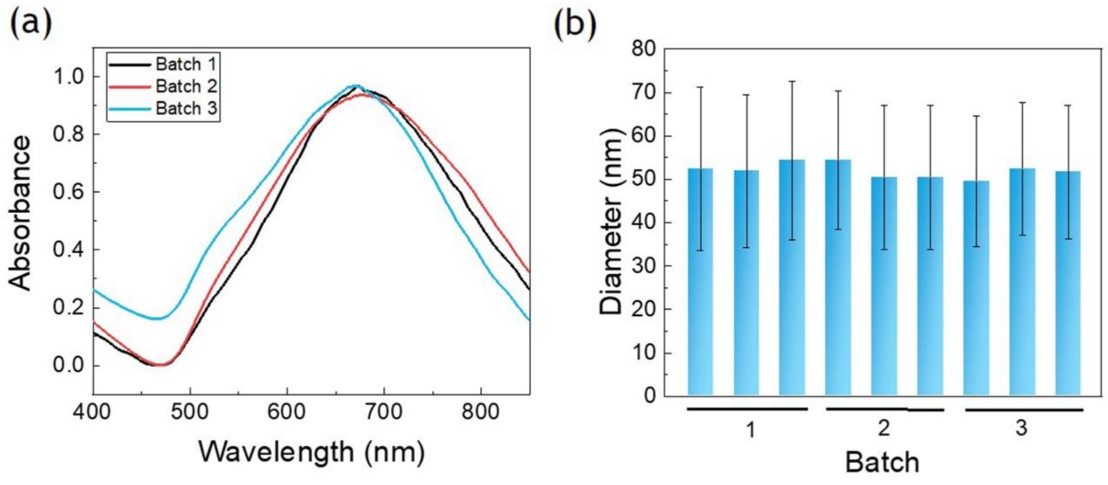

Figure S 2 – Reproducibility between batches. (a) UV-Vis-NIR spectra and (b) hydrodynamic diameter of three independent AuNSs syntheses.

3 Vibrational lines assignment for 4-mercaptobenzoic acid (MBA)

Vibrational lines assignments for MBA are summarised in Table S 2. The areas of the vibrational Raman lines at 1079 cm^{-1} and 1587 cm^{-1} were used to calculate spectral intensity (Figure S 3).

Table S 2 - Vibrational lines assignments for 4-MBA.[6]

Abbreviations: vw: very weak; w: weak; m: medium; s: strong.

Observed bands (cm^{-1})	*Strength*	*Assignment*
630	vw	CCC bending
693	w	CC Stretching
715	w	Ring breathing
845	vw	COO- bending
1013	vw	Ring breathing
1079	s	CC and CS stretching, aromatic ring breathing
1140	vw	CH bending
1177	m	CS binding, CC stretching
1481	w	Aromatic ring bending
1587	s	CC stretching, CH in plane bending
1623	vw	C=O stretching

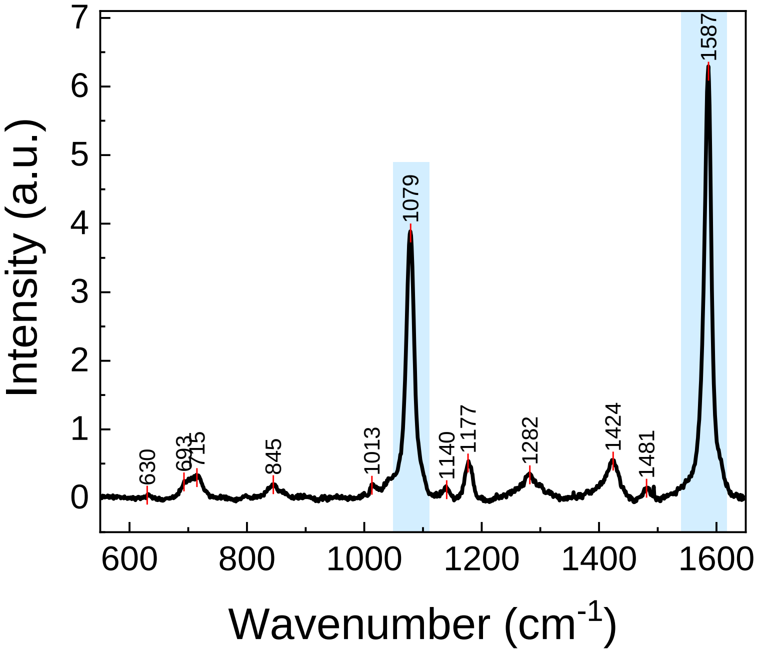

Figure S 3 – Raman spectrum of MBA (2 M) in aqueous solution.

4 Time stability of surfactant-free and functionalised AuNSs

Immediately after synthesis, the colloidal solution of AuNSs exhibited an UV-VIS spectrum with LSPR band maximum centred at 677 nm. The same solution after seven days originated a spectrum with the LSPR band blue-shifted centred at 672 nm (Figure S 4). Therefore, the changes in absorption wavelength maximum (λ_{LSPR}) of two AuNSs batches surfactant free and coated with MBA were analysed to evaluate the stability for subsequent conjugation studies. As seen in Figure S 4, λ_{LSPR} decreases with time and the blue-shift rate varies between batches (also observed by Vega, *et al.*[7]) decreasing in one batch 20 and in the other 80 nm. This change can also be observed by the change of colour in solution from dark blue to purple indicating morphological alterations in the nanostars. This behaviour can be attributed to the loss of AuNSs tips, as reported by Jiang and co-workers[8] and Vega *et al.*[7]. In Vega *et al.* the measurement of the radius at the curvature of the nanostar tips revealed an increase for the surfactant free nanostars and no significant change for the MPA-capped nanostars.[7] Is possible that the Au-S bond from the capping agent and the nanostar limits the diffusion of Au atoms from tips toward the core whereas in the surfactant free AuNSs the gold atoms move driven by the chemical potential difference associated to the respective curvatures.[7], [9]

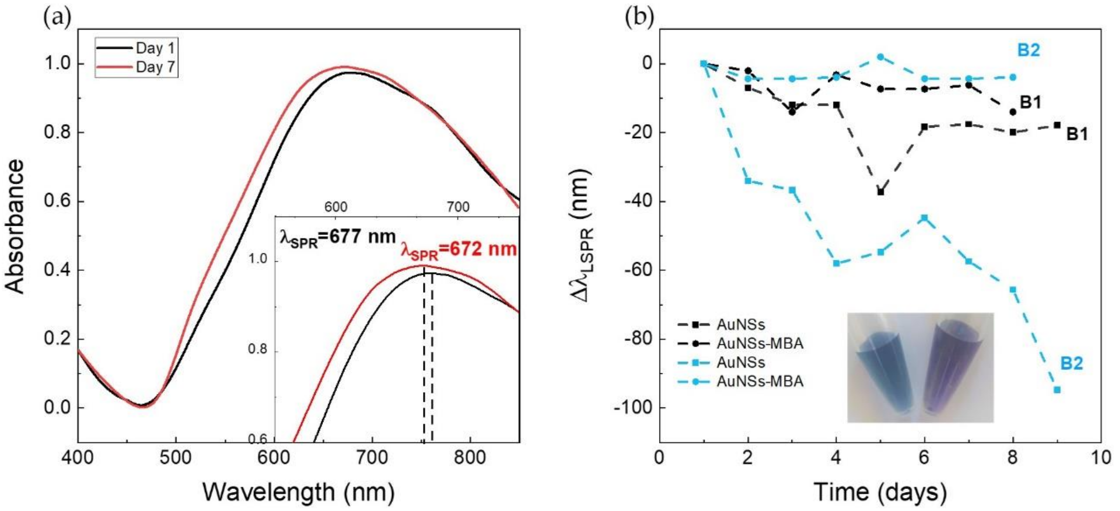

Figure S 4 - Time stability of AuNSs. (a) UV-Vis spectrum after a synthesis of AuNSs (day 1) and after seven days where a blue-shift is perceived indicating the loss of some sharp edges. (b) Shift of the wavelength at maximum absorption for two batches (B1 and B2), both were surfactant free and capped with MBA. The samples were stored at 4 °C and sonicated before each measurement.

The changes in SEM of an AuNSs batch, surfactant free and coated with MBA were analysed to evaluate the stability for subsequent conjugation studies (Figure S 5). However, the images do not confirm the change observed in LSPR band due to the lack of SEM image resolution for such nanometric size particles.

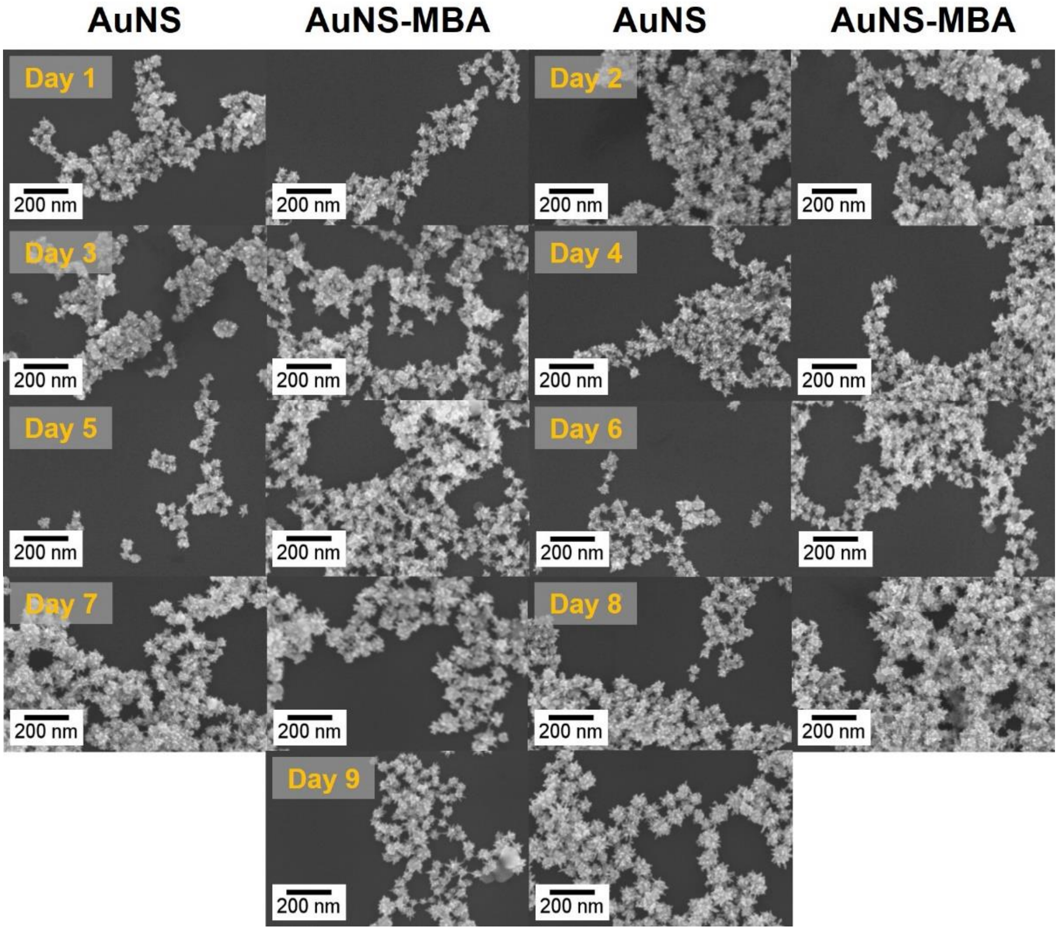

Figure S 5 - SEM micrographs of AuNSs surfactant free and functionalized with MBA throughout nine days.

5 Characterisation of bioconjugates by UV-vis; DLS; ζ-potential; and SEM

To simulate the immunoassay the bioconjugates were incubated with HRP - the respective antigen for the chosen antibody – followed by antibody to bind to HRP, in a sandwich assay. The medium surrounding the nanostructures is responsible for the changes in the refractive index reflecting in LSPR wavelengths shifts.[10] The adsorption/desorption of macromolecules on the surface during bioconjugate formation process was followed by UV-Vis spectroscopy and consistently exhibited a red-shift of the plasmon resonance band relative to the AuNSs-MBA (Figure S 6). The slight broadening of the LSRP band can be a result of AuNSs aggregation or the higher degree of heterogeneity of AuNSs with different loads of antibodies.

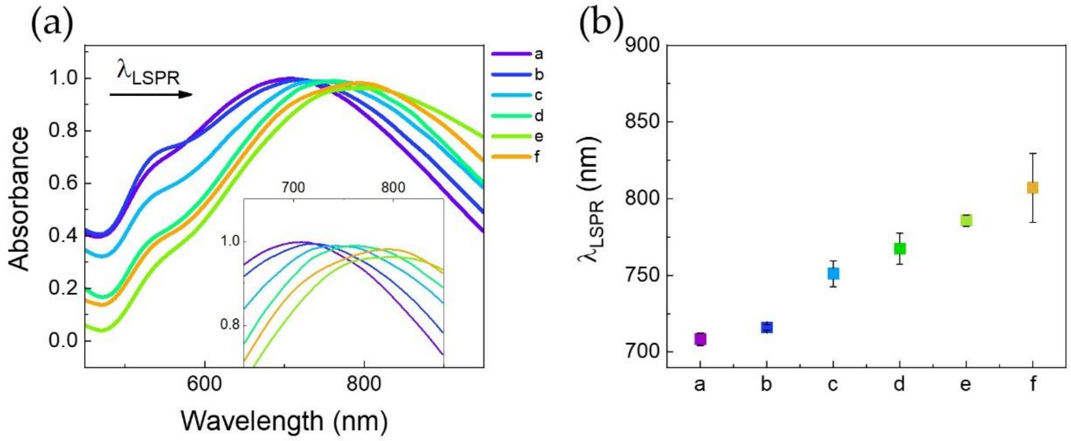

Figure S 6 - (a) UV-Vis spectra and (b) LSPR maximum of bioconjugation process. Normalised optical spectra of bare nanostars (AuNS; a), with functionalisation (AuNS-MBA; b), after antibody conjugation (AuNS-MBA-anti-HRP; c) and blocking with BSA (bioconjugate; d). Followed the bioconjugate production, HRP was added to form (biconjugate-HRP; e) and the sandwich immunoconjugate was completed with the antibody (bioconjugate-anti-HRP; f).

DLS and ζ-potential measurements of the bioconjugates were also obtained to further characterize bioconjugates formation (Figure S 7).

An average hydrodynamic diameter of 67.9 ± 0.4 nm was observed for AuNS-MBA alone (Figure S 7 and Table S 3). Then, the hydrodynamic diameter of the bioconjugates can be related to the amount of protein bound to the AuNSs since by each step of the conjugation, the diameter increases. In fact, for electrostatic conjugation process the subsequent additions of antibody, antigen and antibody again, resulted in shifts of the average hydrodynamic diameter from the value of AuNS-MBA alone, to 82.2 ± 1.3 nm when antibody is bound to MBA, followed by 91.9 ± 1.6 nm when antigen is added and finally, to 135.9 ± 9.3 nm after antibody addition. Although a growing behaviour in hydrodynamic diameter is observed, care must be taken when using DLS data. The conjugation process does not lead to increases that offer peak resolution and with high PDI values as is standard to nanoparticle's systems, highlights the limitation of the method.[11] The dimensions of HRP relating to the conjugate are small (3.0 × 6.5 × 7.5 nm) hence the small of shift for the bioconjugates to bioconjugate-HRP.[12] The arising PDI values after each step of conjugation confirmed the results

observed in UV-Vis spectroscopy. The conjugation steps introduce some aggregation or heterogeneity between samples.

Table S 3 - DLS and Zeta potentials of bioconjugates and following steps toward immunoassay.

		Diameter (nm)	Zeta-Potential (mV)	PDI
	AuNS-MBA	67.9 ± 0.4	-31.6 ± 0.8	0.223
Electrostatic	AuNS-MBA-anti-HRP	75.4 ± 1.3	-25.4 ± 0.8	0.226
	Bioconjugate	82.2 ± 1.3	-24.7 ± 0.9	0.234
	Bioconjugate – HRP	91.9 ± 1.6	-23.8 ± 1.8	0.334
	Bioconjugate – HRP – anti-HRP	135.9 ± 9.3	-22.2 ± 0.8	0.432

Similarly, with the use of AGE, zeta-potential can also be used for evaluating the nanoenvironment. This parameter represents the electrical potential at the interfacial double layer of the conjugates and can be correlated to the amount of protein bound at the nanoparticle surface.

At pH 7, the AuNSs are negatively charged due to physisorbed citrate ions, as confirmed by zeta potential analysis (Figure S 7 and Table S 3). The coverage of the NP surface leads to an electric double layer formation, responsible for preventing aggregation and adsorptive interactions with the walls of the recipients.[13] The global negative charge will interact with the positive charged residues of anti-HRP, consequently the zeta potential increase from -32 mV to -25 mV, consistent with adsorption of antibody on AuNSs surfaces. The following steps of protein addition revealed small decreases in zeta potential stabilizing at -22 mV. The potential observed is indicator of the colloidal suspension stability. This value shows that the colloidal particles are electrically stabilised and hence, can be used in the following assays for determining the positive detection of peroxidase by enzymatic assay and SERS activity.

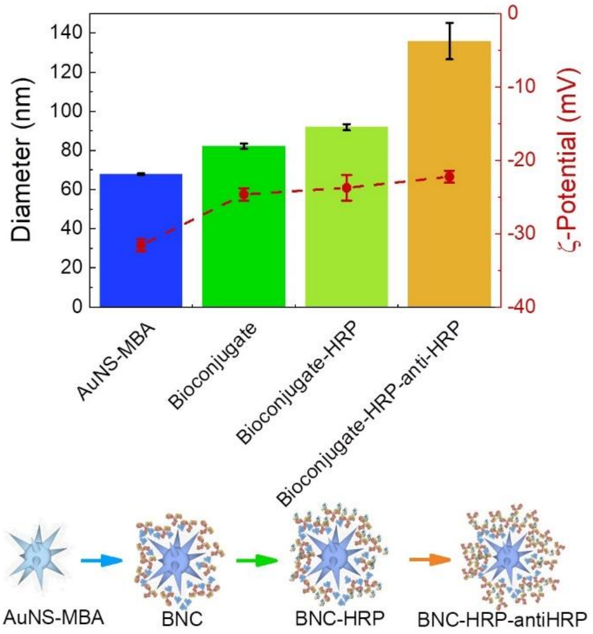

Figure S 7 - Characterization of bioconjugates. *Top*: D_H and zeta-potential of the several steps of bioconjugate formation (error bars are measurements of n = 3 ± standard deviation). *Bottom*: bioconjugation formation scheme.

Scanning electron microscopy has been extremely useful in monitoring morphology and size distribution of nanostructures. Figure S 8 depicts SEM images of AuNSs upon interaction with capping agent, and various additions of protein. SEM confirmed the prevalence of AuNSs morphology during several steps of conjugation and washes.

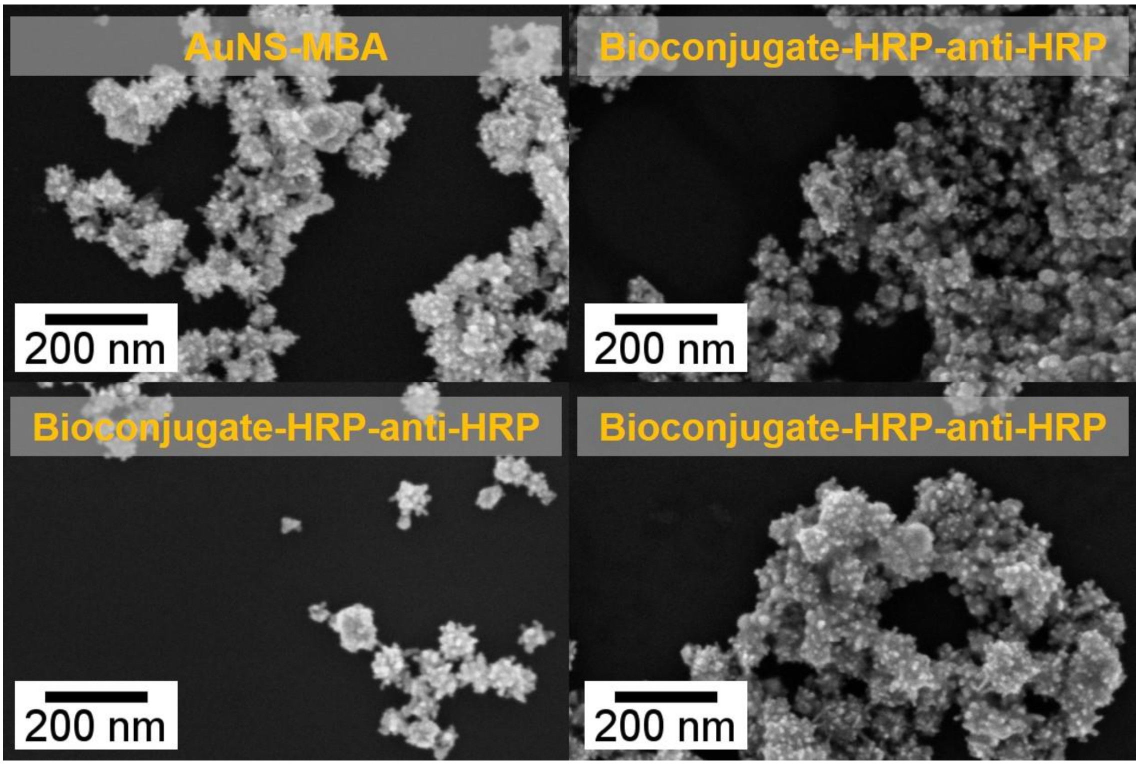

Figure S 8 – Morphological characterization of gold nanostars. SEM images of AuNSs after functionalisation, and conjugation steps. The samples were dispersed in ultrapure water at pH 7. The AuNSs morphology was not lost with increasing amount of protein coupled to the nanoparticles.

6 Peroxidase activity of the bioconjugates supernatants

The bioconjugates were prepared with four different concentrations of antibody, including 211 nM, which was determined by AGE as the maximum amount of antibody covering AuNSs. For each sample tested, the same amount of HRP was added to the bioconjugates and after the incubation time, washed by centrifugation. The supernatant was collected and analysed parallelly with the bioconjugate-HRP samples (Figure S 9). Samples without HRP did not showed any enzymatic activity as expected. As previously reported in literature, BSA is frequently used as a blocking agent to avoid unspecific interactions between the nanoparticle's surface and the analyte.[14] The BSA conjugated with AuNS-MBA can then create steric hindrance avoiding interactions between the AuNS and the HRP. Comparing the supernatants from AuNS-MBA-BSA samples with bioconjugates, it is observable the decrease in activity of the supernatants indicating a higher HRP capture when the antibody is added. The low activity observed in bioconjugates might be related with dissulfide bridges in the antibody. These bridges bind to the nanoparticle leading to a lower load of functional antibody and thus a lower amount of HRP explaining the low activity. The measured activities of supernatants that resulted from the bioconjugates, were similar between samples (despite the increasing concentration of anti-HRP) (Figure S 9). The HRP in the supernatant slightly increased with a decrease in the antibody presented in the bioconjugates (Figure S 9). Nevertheless, the concentration 211 and 422 nM resulted in a lower activity in the supernatant probably due to a higher concentration of HRP being captured by the bioconjugate.

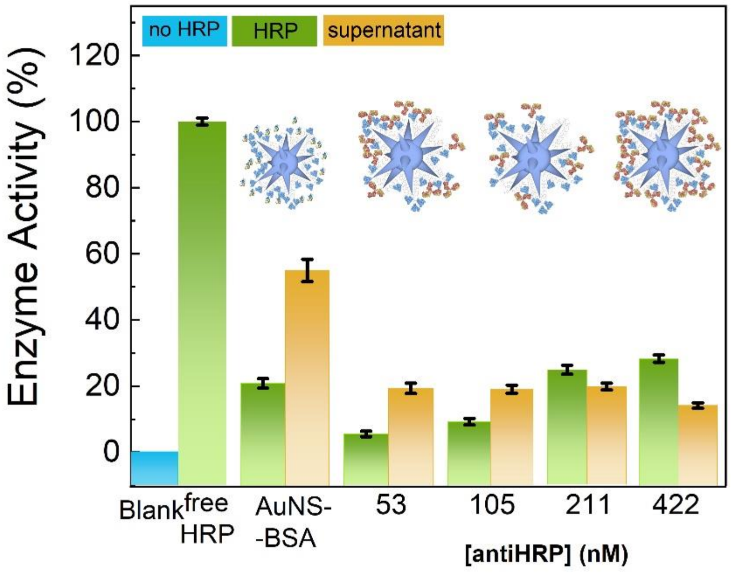

Figure S 9 - Enzymatic assays of bioconjugates. Activities for the bioconjugates and the resulted supernatant: Controls of the enzymatic assay: Blank: assay done with no HRP meaning that the reaction is done with no catalytic agent. Free-HRP: sample consist in the assay with free enzyme. AuNS and AuNS-BSA samples to access the contribution of electrostatic interactions to the assay. Electrostatic bioconjugates with the four concentrations of anti-HRP tested: 53, 105, 211 and 422 nM. The enzymatic activities for bioconjugates with HRP and the resulted supernatant.

7 References

[1] W. Haiss, N. T. K. Thanh, J. Aveyard, and D. G. Ferning, "Determination of size and concentration of gold nanoparticles from extinction spectra," *Anal. Chem.*, vol. 79, no. 17, pp. 4215–4221, 2007.

[2] H. de Puig, J. O. Tam, C.-W. Yen, L. Gehrke, and K. Hamad-Schifferli, "Extinction Coefficient of Gold Nanostars," *J. Phys. Chem. C*, vol. 119, no. 30, pp. 17408–17415, Jul. 2015.

[3] H. Yuan, C. G. Khoury, H. Hwang, C. M. Wilson, G. A. Grant, and T. Vo-Dinh, "Gold nanostars: surfactant-free synthesis, 3D modelling, and two-photon photoluminescence imaging," *Nanotechnology*, vol. 23, no. 7, p. 075102, Feb. 2012.

[4] G. H. Jeong, Y. W. Lee, M. Kim, and S. W. Han, "High-yield synthesis of multi-branched gold nanoparticles and their surface-enhanced Raman scattering properties," *J. Colloid Interface Sci.*, vol. 329, no. 1, pp. 97–102, Jan. 2009.

[5] S. Krishnamurthy, A. Esterle, N. C. Sharma, and S. V. Sahi, "Yucca-derived synthesis of gold nanomaterial and their catalytic potential," *Nanoscale Res. Lett.*, vol. 9, no. 1, pp. 1–9, 2014.

[6] M. Gellner, K. Kömpe, and S. Schlücker, "Multiplexing with SERS labels using mixed SAMs of Raman reporter molecules," *Anal. Bioanal. Chem.*, vol. 394, no. 7, pp. 1839–1844, Aug. 2009.

[7] M. M. Vega, A. Bonifacio, V. Lughi, S. Marsi, S. Carrato, and V. Sergo, "Long-term stability of surfactant-free gold nanostars," *J. Nanoparticle Res.*, vol. 16, no. 11, p. 2729, Nov. 2014.

[8] X. Meng, A. Baride, and C. Jiang, "Ligand Controlled Morphology Evolution of Active Intermediates for the Syntheses of Gold Nanostars," *Langmuir*, vol. 32, no. 26, pp. 6674–6681, 2016.

[9] B. Kingery, H. K. Bowen, and W. D. Kingery, *Introduction to Ceramics*, 2nd ed. John Wiley & Sons, Inc., 1976.

[10] J. Liu and Q. Peng, "Protein-gold nanoparticle interactions and their possible impact on biomedical applications," *Acta Biomater.*, vol. 55, pp. 13–27, Jun. 2017.

[11] S. Bhattacharjee, "DLS and zeta potential – What they are and what they are not?," *J. Control. Release*, vol. 235, pp. 337–351, Aug. 2016.

[12] A. Asokan, J. S. de Ropp, S. L. Newmyer, P. R. Ortiz de Montellano, and G. N. La Mar, "Solution 1 H NMR of the Molecular and Electronic Structure of the Heme Cavity and Substrate Binding Pocket of High-Spin Ferric Horseradish Peroxidase: Effect of His42Ala Mutation," *J. Am. Chem. Soc.*, vol. 123, no. 18, pp. 4243–4254, May 2001.

[13] Z. Wu and D. Li, "Induced-charge electrophoretic motion of ideally polarizable particles," *Electrochim. Acta*, vol. 54, no. 15, pp. 3960–3967, 2009.

[14] S. Puertas *et al.*, "Taking Advantage of Unspecific Interactions to Produce Highly Active Magnetic Nanoparticle−Antibody Conjugates," *ACS Nano*, vol. 5, no. 6, pp. 4521–4528, Jun. 2011.
